# Stable global repertoire architecture masks short-term clonal remodeling in intratumoral TCR repertoires

**DOI:** 10.3389/fimmu.2026.1819997

**Published:** 2026-04-23

**Authors:** Pernille G. Pedersen, Odd L. Gammelgaard, Sofie Traynor, Christina H. Ruhlmann, Aida S. Hansen, Henrik J. Ditzel, Morten F. Gjerstorff, Mikkel G. Terp

**Affiliations:** 1 Unit for Cancer Research, Department of Molecular Medicine, University of Southern Denmark, Odense, Denmark; 2 Department of Oncology, Odense University Hospital, Odense, Denmark; 3 Department of Clinical Research, University of Southern Denmark, Odense, Denmark

**Keywords:** bilateral tumor, clonal remodeling, TCR diversity, TCR repertoire, temporal TCR remodeling

## Abstract

**Background:**

Tumor-infiltrating T cell receptor repertoires are increasingly profiled to assess intratumoral T cell dynamics and inform prognosis and treatment response. Most analyzes rely on aggregate diversity metrics, such as Shannon index or clonality, which describe overall repertoire structure but do not resolve changes in clonotype identity over time. Thus, intrinsic temporal dynamics of intratumoral T cell receptor repertoires during tumor progression remain incompletely defined.

**Methods:**

In a bilateral murine cancer model, one tumor was surgically removed, and the paired tumor was collected 11 days later. The T cell receptor repertoires of these tumors, and of synchronously harvested bilateral tumors from separate control animals, were analyzed using diversity metrics, Morisita–Horn similarity, and clonal tracking approaches.

**Results:**

Time-matched bilateral tumors exhibited highly similar clonotype composition and abundance. In contrast, time-separated tumors showed reduced clonal overlap, increased fractions of private clonotypes, and redistribution of dominant clones. These changes occurred despite preserved global repertoire metrics, including clonotype number, Shannon diversity, and Gini coefficient.

**Conclusion:**

Short-term tumor progression is associated with clear changes in the composition of the intratumoral T cell receptor repertoire, even when overall diversity appears stable. These results suggest that relying solely on global diversity metrics can obscure active clonal remodeling, underscoring the importance of monitoring individual T cell clonotypes to accurately capture intratumoral T cell dynamics over time.

## Introduction

The adaptive immune response is a central determinant of tumor control and therapeutic outcome. Tumor-infiltrating T cells (TILs) recognize tumor-associated antigens through their T cell receptors (TCRs), and the composition of the intratumoral TCR repertoire reflects ongoing antigen-driven selection and immune competition (1–3). Accordingly, high-throughput TCR sequencing has emerged as a clinically relevant tool for prognostic assessment, treatment stratification, and immune monitoring across multiple malignancies (3–7).

Repertoire analyzes typically rely on aggregate diversity metrics, such as the Shannon index or measures of clonality, to quantify overall architecture, summarizing the distribution of clones within a sample and often serving as indicators of repertoire stability (8, 9). High diversity reflects broad clonal representation, whereas high clonality indicates dominance of expanded T cell populations (10, 11). Although widely applied, these metrics summarize repertoire structure and do not resolve whether individual clonotypes expand, contract or are replaced over time. Stable diversity values may therefore conceal substantial changes in clonal identity.

Most longitudinal studies of TCR repertoire dynamics have been conducted in the context of therapeutic intervention. Chemotherapy, radiotherapy, immune checkpoint blockade, and targeted therapies can induce selective expansion, redistribution or replacement of T cell clones associated with clinical response (12–16). These observations demonstrate that therapeutic perturbation can reshape the repertoire. However, the intrinsic temporal dynamics of intratumoral TCR repertoires during tumor progression in the absence of treatment remain less well characterized.

Tumor growth is accompanied by evolving antigen presentation, clonal competition, and continuous recruitment and selection of T cells (17, 18). Such processes could drive clonal remodeling even when global diversity metrics remain stable. However, the extent to which short-term tumor progression alone drives measurable clonal remodeling, particularly at the level of clonotype identity rather than aggregate diversity, remains unclear. Disentangling temporal evolution from spatial heterogeneity is therefore critical for interpreting single-biopsy analyzes and longitudinal immune profiling.

Here, we used a bilateral model to control for host background and tumor antigenicity, while enabling paired time-matched and time-separated comparisons within individual animals. We show that time-matched bilateral tumors exhibit highly conserved intratumoral TCR repertoire architecture within the same host, indicating strong spatial concordance. In contrast, time-separated tumors display reduced clonal overlap and considerable redistribution of individual clonotypes over an interval of only 11 days, despite preserved global diversity metrics. These findings demonstrate that short-term tumor progression is accompanied by marked clonal restructuring that may be masked by stable aggregate repertoire measures.

## Materials and methods

### Mice

All animal experiments were performed at the animal facility at University of Southern Denmark and approved by the Animal Experiments Inspectorate of the Ministry of Food, Agriculture and Fisheries of Denmark. Female BALB/c mice aged between 7 to 9 weeks were obtained from Charles River Laboratories. Mice were acclimatized for at least one week before initiation of experiments and were housed with ad libitum access to food and water under specific pathogen-free conditions. Mice were euthanized by cervical dislocation.

### Cell culture and inoculation

The murine colon cancer cell line CT26.CL25 (here referred to as CT26) were obtained from American Type Culture Condition (ATCC, CRL-2639), and cultured in Roswell Park Memorial Institute 1640 Medium (RPMI+GlutaMAX™, Gibco), supplemented with 10% fetal bovine serum (FBS, Sigma), 4.5 g/L Glucose Solution (Gibco), 1 nM Sodium Pyruvate (Gibco), 10 nM HEPES Buffer Solution (Gibco), 0.1 nM non-essential amino-acids (NEAA, Gibco) and 0.5 mg/mL G418 Sulfate (Corning). The cells were kept in humidified atmosphere with 5% CO_2_ at 37 °C. To establish bilateral tumors, mice were shaved and inoculated subcutaneously with 0.5 x 10^6^ CT26 cancer cells in each flank in ECM gel from Engelbreth-Holm-Schwarm murine sarcoma (Sigma-Aldrich).

### Time-matched group

Mice were inoculated as previously described. After bilateral tumors had formed, both tumors were simultaneously harvested after euthanasia 13 days after inoculation. Tumors were snap-frozen in liquid nitrogen and stored at -80 °C before further processing. Three mice were included in this group.

### Time-separated group

Mice were inoculated as previously described. When bilateral tumors had reached a size of approximately 4–5 mm in diameter, mice were anesthetized, and one tumor was surgically excised (referred to as the “early tumor”). Wounds were closed with sterilized clips, and mice were given 5 mg/kg carprofen and 0.1 mg/kg burprenorphin subcutaneously as analgesic treatment. The remaining tumor was left to grow and was harvested 11 days after the surgery after euthanasia (referred to as the “late tumor”). Tumors were snap-frozen in liquid nitrogen and stored at -80 °C before further processing. Three mice were included in this group.

### Decitabine treatment groups

Mice were inoculated and one tumor surgically removed as previously described (referred to as the “early tumor”). Three days after removal of one of the tumors, mice were randomized into two treatment groups, that were treated with 0.5 mg/kg or 2 mg/kg decitabine (DAC, SelleckChem, S1200) administered intraperitoneally for five consecutive days. Four days after the last treatment, the remaining tumor was harvested after euthanasia (referred to as the “late tumor”). Tumors were snap-frozen in liquid nitrogen and stored at -80 °C before further processing. Three mice were included in each treatment group. The DAC treatment groups were compared to the time-separated group, acting as an untreated control (referred to as “vehicle”), following the same interval between tumor inoculation, surgery and tumor harvest.

### Tissue processing

RNA was purified from whole tumors using TriReagent (Sigma) and EconoSpin Columns (Epoch Life Science). Tumors were homogenized by adding 2.8 mm Zirconium beads (Precellus) to the tissue, and homogenization was carried out using a Precellus Homogenizer run at 3 x 15 seconds at 6500 rpm. RNA quality was assessed by TapeStation 4150 (Agilent Technologies) following manufacturer’s instructions.

### Bulk TCR sequencing

TCR*α* and TCR*β* receptor libraries covering the complementarity determining region 3 (CDR3) were prepared using SMART-Seq Mouse TCR (with UMIs) (Takara, 634815) and SMARTer RNA Unique Dual Index Kit (Takara, 634451) following manufacturer’s instructions. Library quality was assessed using TapeStation 4150 following manufacturer’s instructions. The TCR libraries were sequenced on the Illumina NextSeq 2000 platform using read lengths of 150 bp read 1, 8 bp i7 index, 150 bp read 2, 8 bp i5 index and 20% PhiX. The TCR libraries were sequenced to a minimum depth of 25x10^6^ reads (manufacturer’s recommendation: minimum 15x10^6^ reads). TCR reads were pre-processed using the Cogent NGS Immune Profiler Software v2.0 (Takara) where data pre-processing, UMI based analysis and clonotype calling were performed.

### Data analysis

The output from the Cogent NGS Immune Profiler was further analyzed in RStudio (version 2025.05.0 + 496) using the software packages immunarch (19) and vegan (20). Data analysis of *α*- and *β*-chains were performed separately using identical pipelines. The data was initially pre-filtered to remove non-functional sequences, excluding TCRs encoding stop codons. In addition, low-frequency clonotypes found with only a single read were excluded to reduce the impact of sequencing errors and PCR artefacts. After data pre-processing and filtering, we retained an average of 198,734 reads (range: 26,623-846,655) for TCR*α* repertoires, and 310,963 (range: 63,291-887,567) for TCR*β* repertoires. We defined unique clonotypes as TCRs sharing the same amino acid sequence and the same V-, and J-gene segment. To ensure proper comparison across samples with differing sequencing depth, the data was downsampled to the smallest repertoire size using the *repSample* function of the immunarch package using the arguments:*.methods=downsample,.n=NA*, performing downsampling without any probabilistic simulation. The Morisita-Horn index was used to evaluate repertoire overlap, which measures the overlap of identical clones weighted by their abundancies (10, 21). Repertoire diversity was calculated using the normalized Shannon index, that takes both richness and evenness into account (10, 21) and Gini co-coefficient, that quantifies the evenness of the repertoires (10). The number of clonotypes and Morisita-Horn index was calculated using the *repExplore* and *repOverlap* functions of the immunarch package, respectively. The normalized Shannon Diversity index was calculated using the *diversity* function of the vegan package and Gini coefficient using the *repDiversity* function of the immunarch package. When comparing TCR repertoires between “early” and “late” tumors, private clones were defined as those detected exclusively in one tumor within the same mouse, while shared clones were defined as those present in both tumors within the same mouse.

### Statistics

Data was visualized in Rstudio and GraphPad Prism (version 10.4.2), and statistical analysis performed using GraphPad Prism. Differences between groups were assessed using paired or unpaired t-tests as appropriate with the specific tests indicated in the corresponding figure legends. Given the small sample size (n=3 per group), parametric tests were employed as an approximation to compare group means. Accordingly, statistical tests were considered exploratory in nature.

## Results

### Simultaneously harvested bilateral tumors are synchronized at the clonal level

**Figure 1:**
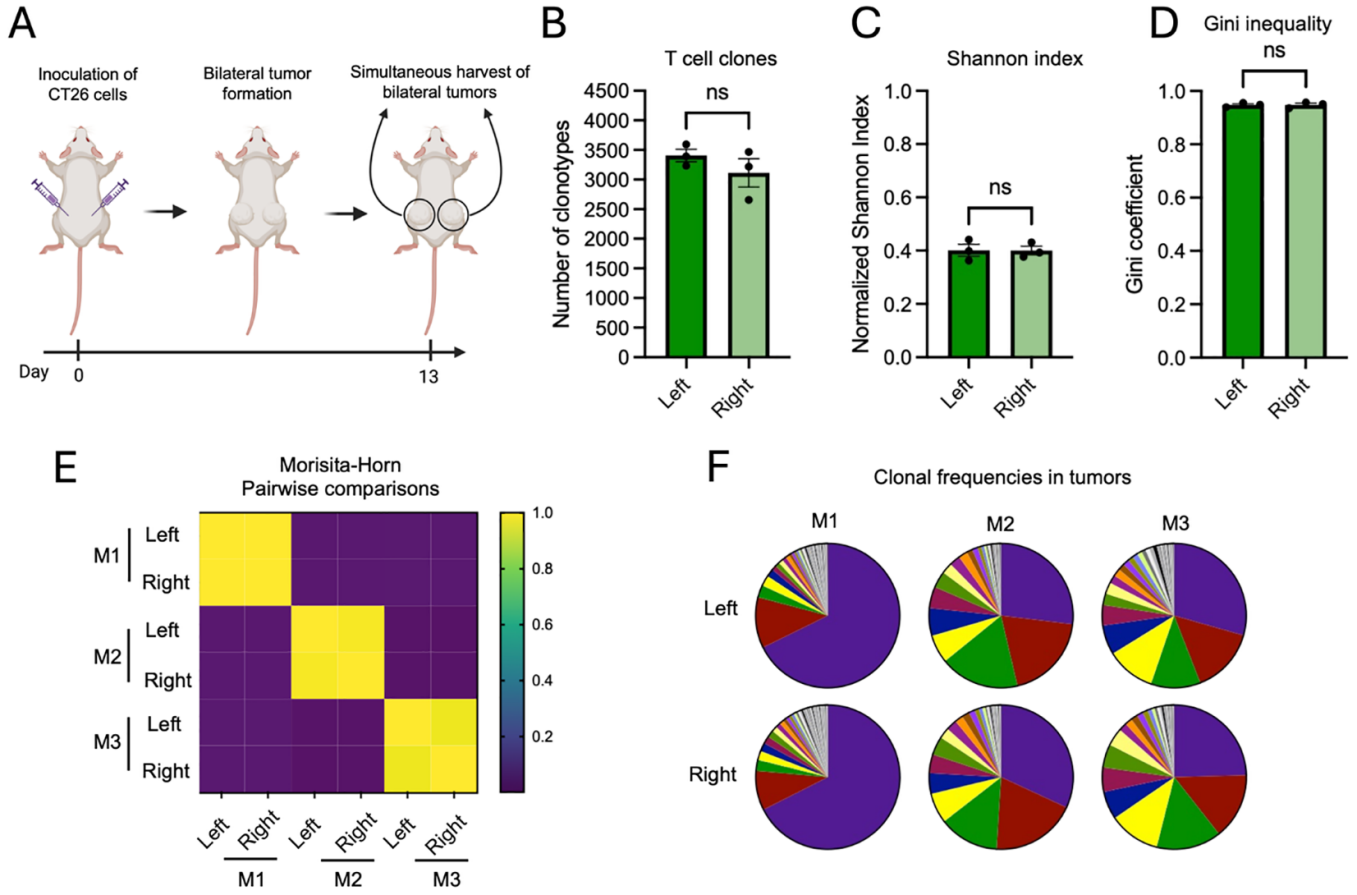
Limited intertumoral divergence of TCRβ repertoires in synchronously established bilateral tumors. **(A)** Schematic outline of the experimental setup. Mice were inoculated subcutaneously with CT26 cells in both flanks and tumors were harvested simultaneously on day 13 for TCR sequencing. **(B–D)** Total clonotype number **(B)** repertoire diversity (normalized Shannon index) **(C)**, and clonality (Gini coefficient) **(D)** were comparable between left and right tumors. **(E, F)** Morisita-Horn similarity matrix **(E)** and clonal frequency distributions **(F)** comparing paired left and right tumors from three independent mice (M1-M3). Repertoires of contralateral tumors within each mouse were largely indistinguishable. Data are presented as mean +/- SEM. Statistical difference was assessed using a paired t-test. ns, p > 0.05. N = 3 mice per group.

To establish a baseline for assessing longitudinal repertoire dynamics, we first evaluated TCR similarity between time-matched bilateral tumors from untreated mice (**Figure 1A**). This model enables paired analysis within individual animals while minimizing inter-individual variability (22). At the global level of repertoire architecture, TCR*β* repertoires from bilateral tumors within the same mouse were highly similar, with comparable clonotype numbers and diversity indices (**Figures 1B–D**).

Similar patterns were also observed for TCR*α* repertoires (**Supplementary Figures 1A–D**). To directly assess clonal overlap, we performed Morisita-Horn similarity analysis. Paired tumors within the same mouse exhibited substantial overlap (**Figure 1E**), whereas repertoires were clearly distinct across different mice. Consistently, the distribution of intratumoral clonotypes were closely matched between bilateral tumors (**Figure 1F**), indicating conserved clonal hierarchy and expansion dynamics. Similar overlap patterns were also observed for TCR*α* repertoires (**Supplementary Figures 1E, F**). Collectively, these findings demonstrate that time-matched bilateral tumors display highly conserved intratumoral TCR architecture at a given timepoint, validating this model for subsequent longitudinal analysis.

### Short-term tumor progression is associated with clonal remodeling despite preserved global repertoire structure

**Figure 2:**
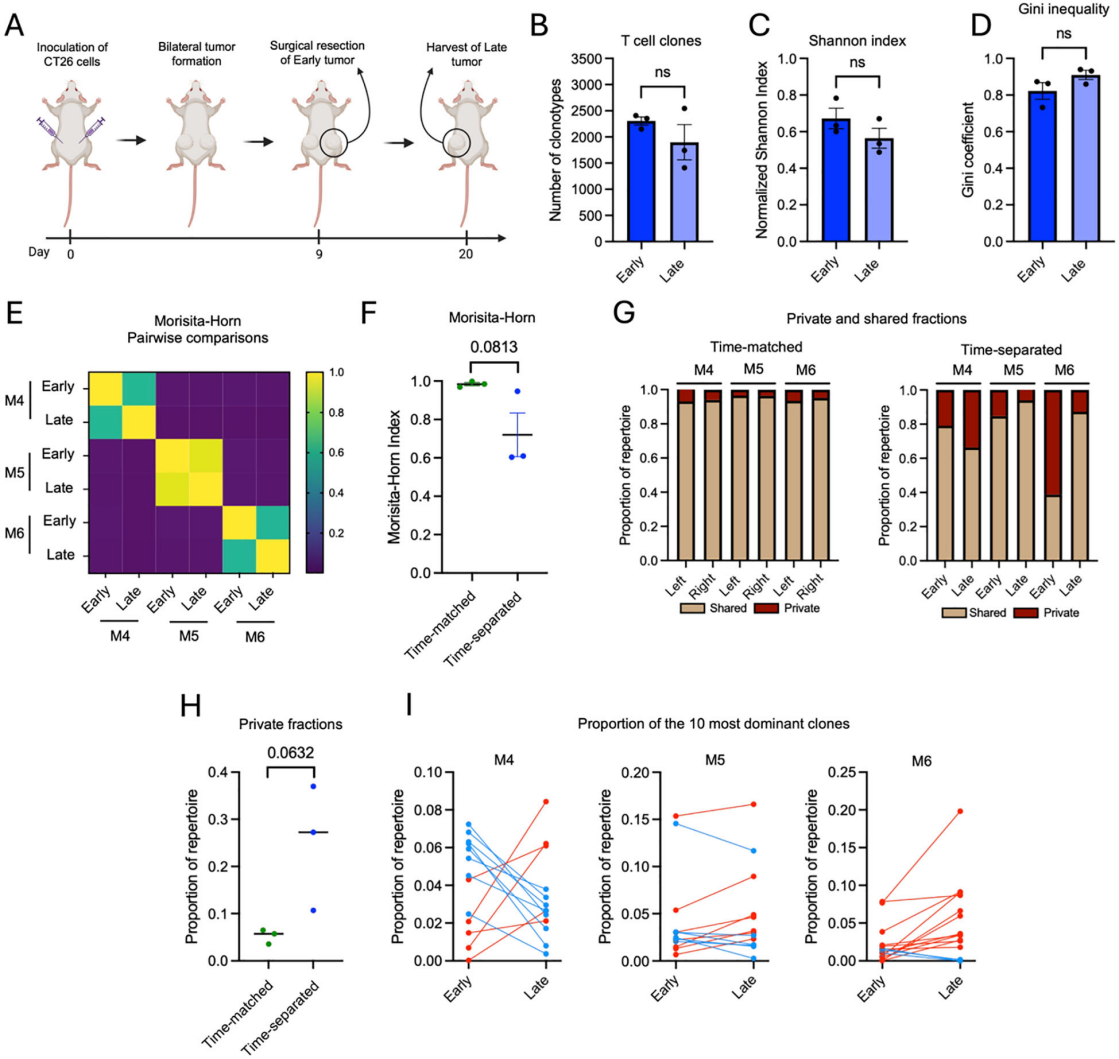
Short-term tumor progression induces clonal turnover while overall TCRβ repertoire structure remains stable. **(A)** Schematic outline of the experimental setup. Mice were inoculated subcutaneously with CT26 cells in both flanks at day 0. One tumor was surgically resected at day 9, and the remaining tumor were harvested at day 20. Early and late tumors were subjected to TCR sequencing. **(B–D)** Total clonotype number **(B)**, repertoire diversity (normalized Shannon index **(C)**, and clonality (Gini coefficient) **(D)** did not differ between early and late tumors. **(E, F)** Morisita-Horn similarity analysis comparing early and late tumors from three independent mice (M4-6) **(E)** and time-matched and time-separated groups **(F)**. Similarity was consistently reduced in time-separated tumors compared with time-matched controls, indicating temporal repertoire divergence. **(G)** Proportion of shared and private clonotypes in time-matched versus time-separated tumors. **(H)** Proportion of private clonotypes in time-matched and time-separated tumors. **(I)** Temporal dynamics of the 10 most dominant clonotypes within each mouse, demonstrating expansion and contraction of individual clones between early and late tumors. Blue lines denote decreasing and red lines increasing clonotype frequencies. Data are presented as mean ± SEM. Statistical significance was assessed using paired **(B–D)** or unpaired **(F, H)** t-tests. ns, p > 0.05. N = 3 mice per group.

Having established strong concordance between simultaneously harvested tumors, we next assessed how intratumoral TCR repertoires evolved over time. To enable a time-separated comparison within individual animals, one tumor was surgically removed at an early timepoint, while the contralateral tumor was harvested 11 days after (**Figure 2A**). We first assessed whether overall TCR*β* repertoire structure changed during this interval. The total number of detected clonotypes was similar between early and late tumors (**Figure 2B**). Likewise, normalized Shannon index and Gini coefficient did not differ significantly (**Figures 2C, D**). Thus, at the level of global diversity and evenness, the repertoires appeared largely stable over time. Similar patterns were observed for TCR*α* repertoires (**Supplementary Figures 2A–D**). We then examined clonal overlap directly. Morisita-Horn analysis showed that early and late tumors within the same mouse were less similar than simultaneously harvested tumors (**Figure 2E**). This difference was confirmed when comparing Morisita-Horn indices between the time-matched and time-separated settings (**Figure 2F**), indicating reduced repertoire overlap over time. To further characterize this divergence, we analyzed the proportion of shared and private clones. Our analysis revealed that time-separated tumors contained a lower fraction of shared clones and a higher fraction of private clones as compared to time-matched tumors (**Figures 2G, H**), consistent with measurable clonal turnover during short-term tumor progression. Finally, we tracked dominant clonotypes across timepoints showing that while many clones remained detectable at both timepoints, their relative abundances changed. Some clones expanded, while others diminished (**Figure 2I**), indicating a redistribution within the repertoire. The same repertoire overlaps and changes at the clonal level was observed for TCR*α* repertoires (**Supplementary Figures 2E–I**). Together, these results show that short-term tumor progression is associated with measurable clonal remodeling, even though global diversity metrics remain stable.

### Longitudinal clonal remodeling is reproducible across experimental conditions

**Figure 3:**
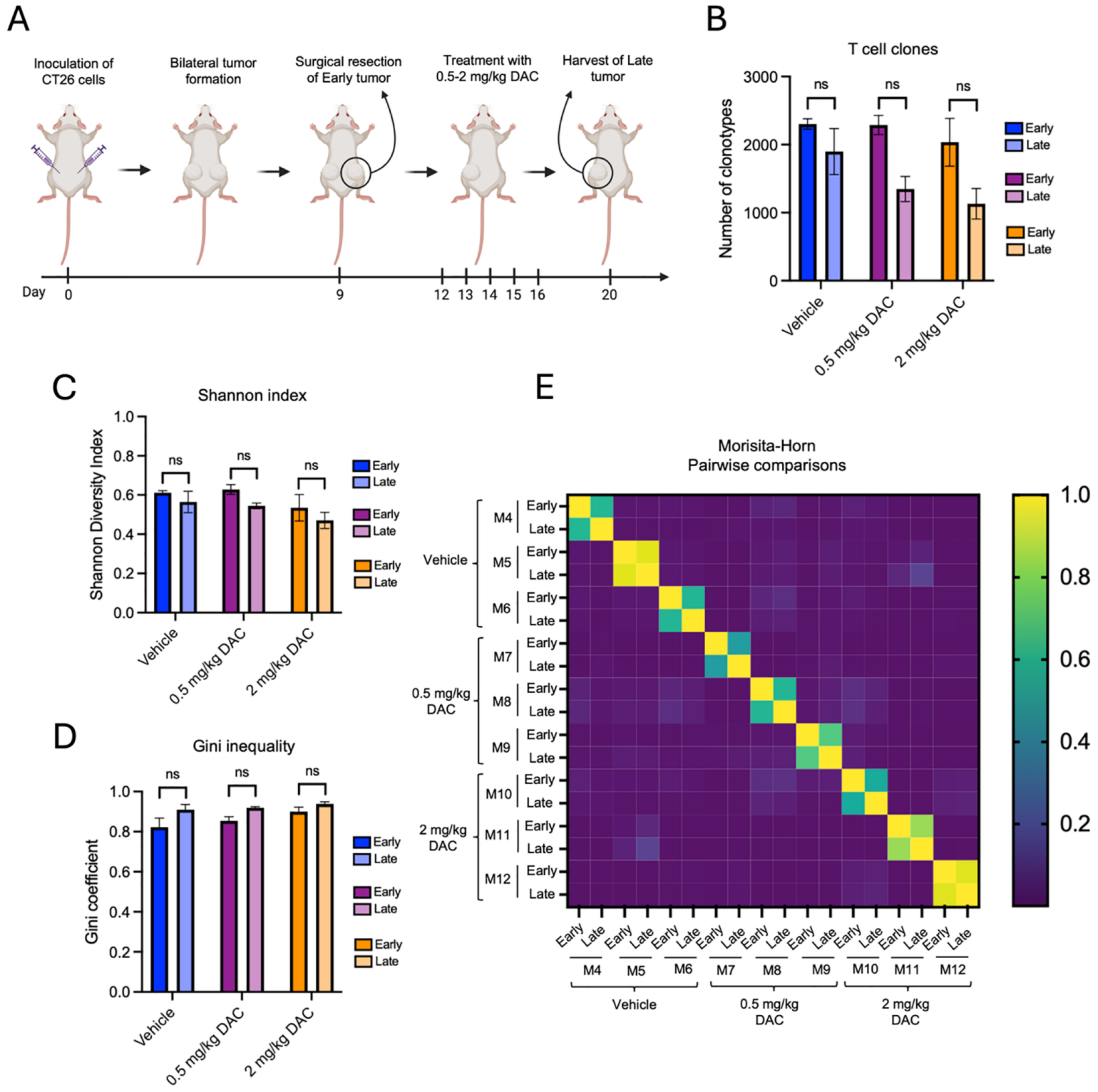
Longitudinal remodeling of TCRβ repertoires is consistent across experimental conditions. **(A)** Schematic outline of the experimental setup. CT26 cells were inoculated subcutaneously in both flanks. One tumor was surgically resected at day 9 and the remaining tumor was harvested at day 20 following treatment (vehicle or DAC (0.5 mg/kg, or 2 mg/kg),). Early and late tumors were subjected to TCR sequencing. **(B–D)** Total clonotype number **(B)**, repertoire diversity (normalized Shannon index **(C)**, and clonality (Gini coefficient) **(D)** did not differ between early and late tumors within each treatment group. **(E)** Morisita–Horn similarity matrix comparing paired early and late tumors from three independent mice across treatment groups (M4-6 & M7-9 & M10-12). Temporal repertoire divergence was observed within individual mice irrespective of treatment. Data are presented as mean ± SEM. Statistical significance was assessed using paired t-tests. ns, p > 0.05. N = 3 mice per group. The vehicle group corresponds to the same mice presented in **Figure 2**.

To determine whether pharmacologic intervention altered the observed longitudinal clonal remodeling, we examined TCR repertoires in mice treated with two doses of the DNA methyltransferase inhibitor DAC or vehicle following early tumor resection (**Figure 3A**). Across all treatment groups, global TCR repertoire metrics remained comparable between early and late tumors, with clonotype numbers, normalized Shannon index and Gini coefficient showing no consistent differences over time (**Figures 3B–D**, **Supplementary Figures 3B–D**). We next assessed repertoire overlap and found that Morisita-Horn indices between early and late tumors were similarly reduced across vehicle and both DAC-treated groups (**Figure 3E**, **Supplementary Figures 3E and 4A**). Consistent with this, the proportion of shared clonotypes remained lower in late tumors across all groups, with a corresponding increase in private clones (**Supplementary Figures 4B, C**). Together, these findings demonstrate that short-term longitudinal clonal remodeling is reproducibly observed across independent cohorts and treatment conditions.

## Discussion

In this study, we examined how intratumoral TCR repertoires evolve over time. Time-matched bilateral tumors displayed strong concordance in clonotype composition and abundance, as previously described (22). In contrast, tumors within the same host separated by an 11-day interval showed reduced overlap and reorganization within the shared clonotype compartment, as well as an increased proportion of private clonotypes. Notably, these temporal differences were observed despite largely preserved global diversity metrics, indicating that stable aggregate measures can mask underlying clonal remodeling. Together, these findings provide an exploratory but consistent demonstration that short-term tumor progression is accompanied by measurable restructuring at the level of clonotype identity.

Spatial heterogeneity of TIL repertoires has been reported across anatomically distinct lesions and multi-region tumor samples in human cancer (23, 24). Such divergence is often attributed to differences in tissue context, antigen dynamics, or microenvironmental selection pressures between anatomical sites. In contrast, the bilateral subcutaneous model provides a controlled setting in which tumor antigenicity, host background, and local tissue environment are largely shared. Within this framework, divergence in clonal composition emerged primarily along a time-dependent trajectory, rather than a spatial trajectory, suggesting that in this controlled setting, temporal dynamics may contribute more to intratumoral TCR divergence than spatial variation.

Longitudinal changes in TCR repertoires have been described in the context of immunotherapy, where treatment-induced expansion or contraction of specific clones can reshape the repertoires (25–27). While some degree of clonal fluctuation during tumor progression is biologically anticipated, our data indicate reproducible divergence over a relatively short interval in the absence of therapeutic intervention. The magnitude of remodeling observed over only 11 days suggests a potentially important role for longitudinal dynamics in shaping intratumoral TCR architecture. Notably, these changes occurred without major differences in commonly used diversity measures such as Shannon index and Gini coefficient. These metrics summarize the overall distribution of clones within a sample and are often interpreted as indicators of repertoire stability (8, 9). Importantly, stable global diversity metrics did not reflect clonal stability, but masked substantial turnover at the level of individual clonotypes. This suggests that temporal heterogeneity represents an additional and underappreciated dimension of intratumoral immune heterogeneity, encompassing turnover of clonotype identity over time that is not captured by static diversity metrics. Analyzes based solely on global diversity metrics may therefore overlook ongoing clonal remodeling. The increased contribution of private clonotypes observed in temporally separated tumors may reflect either a broadening of the TCR repertoire or, alternatively, a more fragmented and potentially less effective response characterized by continual recruitment of new, low-frequency clones. The current data do not assess these possibilities, and the functional relevance of these dynamics remains to be determined.

Several non-mutually exclusive mechanisms may contribute to the observed longitudinal remodeling, including changes in antigen presentation, differential clonal fitness, and microenvironmental selection (28–31). However, as the present study is based on bulk TCR sequencing, we cannot determine whether these dynamics reflect tumor-reactive-, bystander-, or functionally distinct T cell populations, nor link individual clonotypes to antigen specificity. Accordingly, we cannot distinguish whether clonal turnover is driven by quantitative changes in antigen load or qualitative shifts in the neoantigen landscape, such as loss or emergence of clonal or subclonal antigens. Still, the observed patterns are consistent with ongoing antigen-driven and microenvironmental selection, and may reflect T cell responses adapting to evolving antigenic targets.

The dynamic nature of intratumoral TCR remodeling observed here may also have translational implications. Many translational studies rely on single baseline biopsies to characterize TIL repertoires and to derive diversity-based biomarkers predictive of therapeutic response (12, 32–34). If substantial clonal redistribution and partial clonal turnover occur over short intervals even in the absence of therapy, such measurements may represent a temporally constrained snapshot of an evolving immune landscape. This suggests that static diversity-based metrics may be insufficient to capture the full extent of intratumoral immune heterogeneity. This temporal variability may also have implications for therapeutic strategies, relying on stable antigen targets, such as neoantigen-based vaccines or TCR-engineered T cell therapies. If the TCR repertoire undergoes reorganization even over short time intervals, this raises the possibility that the underlying neoantigen landscape is similarly dynamic. Consequently, therapeutic targets identified at baseline may be lost, diluted, or no longer dominant by the time of treatment, potentially limiting the effectiveness of strategies that rely on stable antigen targets. This suggests that longitudinal sampling strategies may improve identification and tracking of relevant immune targets over time. However, further studies are still required to determine the extent to which these dynamics impact such therapies.

We acknowledge that our study has limitations. In particular, the small sample size (n=3 mice per group) constrains statistical power and underscores the exploratory interpretation of our findings. Second, the study was conducted in a single murine model, and the extent to which these results generalize to other tumor types or to human disease remains to be determined. Thus, further validation in larger cohorts and diverse experimental models will be necessary. Further resolution of clonotype-specific properties will require integration of functional assays and single-cell approaches, such as paired TCR and transcriptomic profiling, to more precisely define the properties of dynamically changing clonotypes. Finally, our study was restricted to a predefined longitudinal interval, which may not fully capture the kinetics of T cell clonal evolution.

In summary, we demonstrate that short-term tumor progression is accompanied by measurable intratumoral TCR repertoire remodeling, encompassing both partial clonal replacement and redistribution within the shared repertoire, despite stable global diversity metrics. These findings support the relevance of clonal-level analyzes for accurately capturing immune dynamics and suggest that apparent repertoire stability may mask ongoing clonal evolution.

## Data Availability

The NGS data generated during the study is available in the Gene Expression Omnibus, GSE repository, accession number GSE328312.
