# Supplementary material for: Stable global repertoire architecture masks short-term clonal remodeling in intratumoral TCR repertoires
Source: Front Immunol. 2026 Apr 23;17:1819997. doi: 10.3389/fimmu.2026.1819997 (PMC13149252; doi:10.3389/fimmu.2026.1819997)
Supplement: Supplementary file 1 [file DataSheet1.pdf]

Supplementary Figures

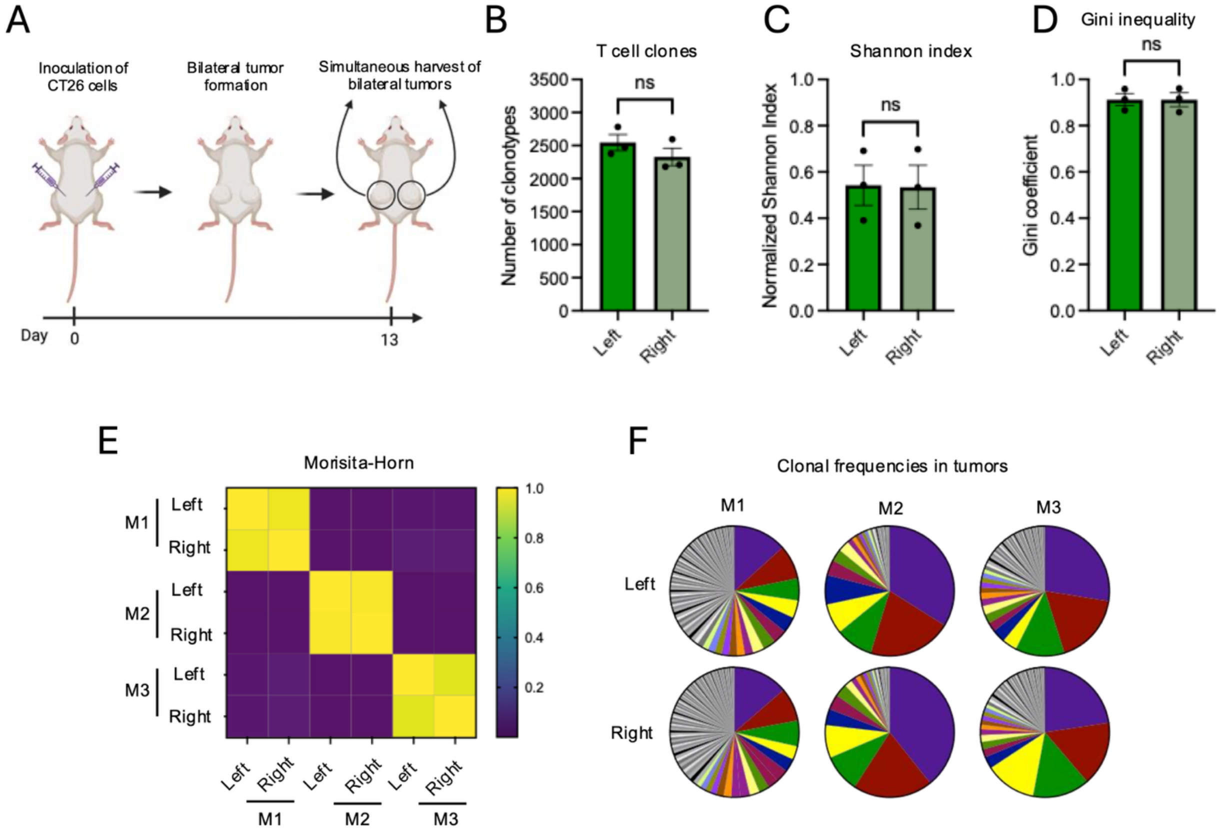

Supplementary Figure 1. TCRα repertoires of time-matched tumors are highly similar. A) Workflow of the time-matched setting. Mice were inoculated subcutaneously with CT26 cells in both flanks. Tumors were harvested on day 13 for TCR sequencing. **B–D)** Total clonotype number *(B)*, repertoire diversity (normalized Shannon index *(C)*, and clonality (Gini coefficient) *(D)* were comparable between left and right tumors. **E)** Morisita–Horn similarity matrix comparing paired left and right tumors from three independent mice (M1–M3). Repertoires of contralateral tumors within each mouse were largely indistinguishable. **F)** Clonal frequency distributions for each tumor within individual mice. Data are presented as mean ± SEM. Statistical significance was assessed using paired t-tests. ns, $p > 0.05$. N = 3 mice per group.

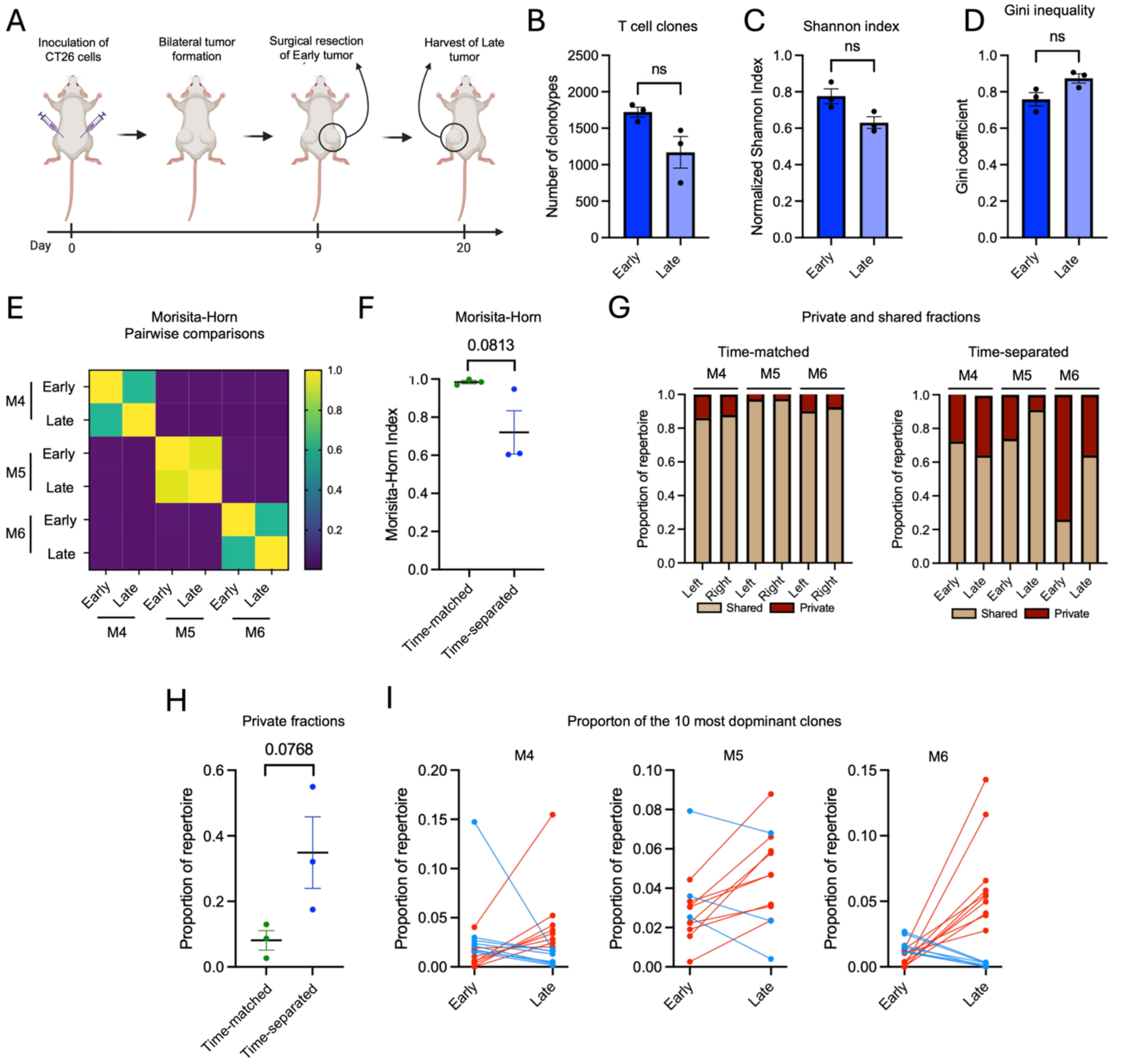

Supplementary Figure 2. Temporal clonal remodeling in time-separated tumors while overall repertoire structure remains stable. A) Schematic outline of the time-separated setting. CT26 cells were inoculated subcutaneously in both flanks. One tumor was surgically resected at day 9, and the remaining tumor were harvested at day 20. Early and late tumors were subjected to TCR sequencing. **B–D)** Total clonotype number *(B)*, repertoire diversity (normalized Shannon index *(C)*, and clonality (Gini coefficient) *(D)* did not differ between early and late tumors. **E–F)** Morisita–Horn similarity analysis comparing early and late tumors from three independent mice (M4-6). Similarity was reduced in time-separated tumors compared with time-matched controls. **G)** Proportion of shared and private clonotypes in time-matched versus time-separated tumors. **H)** Proportion of private clonotypes in time-matched and time-separated tumors. *I)* Temporal dynamics of the 10 most dominant clonotypes within each mouse. Blue lines denote decreasing and red lines increasing clonotype frequencies. Data are presented as mean ± SEM. Statistical significance was assessed using paired t-tests. ns, $p > 0.05$. N = 3 mice per group.

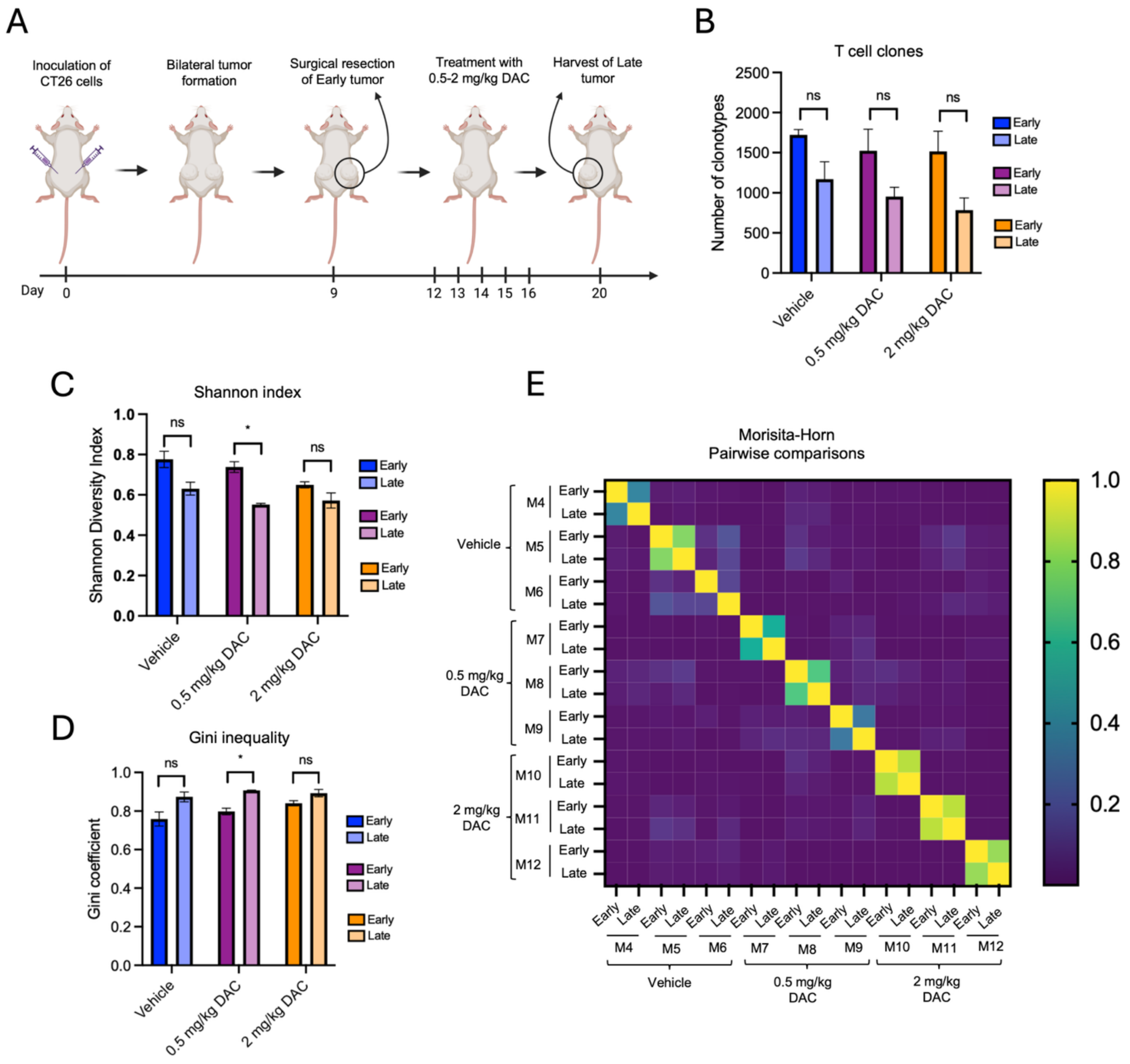

Supplementary Figure 3. Temporal clonal remodeling across DAC treatment conditions. A) Schematic outline of the experimental setup. CT26 cells were inoculated subcutaneously in both flanks. One tumor was surgically resected at day 9 and the remaining tumor was harvested at day 20 following treatment (vehicle or DAC (0.5 mg/kg or 2 mg/kg)). Early and late tumors were subjected to TCR sequencing. **B–D)** Total clonotype number *(B)*, repertoire diversity (normalized Shannon index, *(C)*, and clonality (Gini coefficient, *(D)* did not differ between early and late tumors within each treatment group. **E)** Morisita–Horn similarity matrix comparing paired early and late tumors from three independent mice across treatment groups (M4-6 & M7-9 & M10-12). Temporal repertoire divergence was observed within individual mice irrespective of DAC dose. Data are presented as mean ± SEM. Data are presented as mean ± SEM. Statistical significance was assessed using paired t-tests. ns, $p > 0.05$. N = 3 mice per group. The vehicle group corresponds to the same mice presented in Figure 2

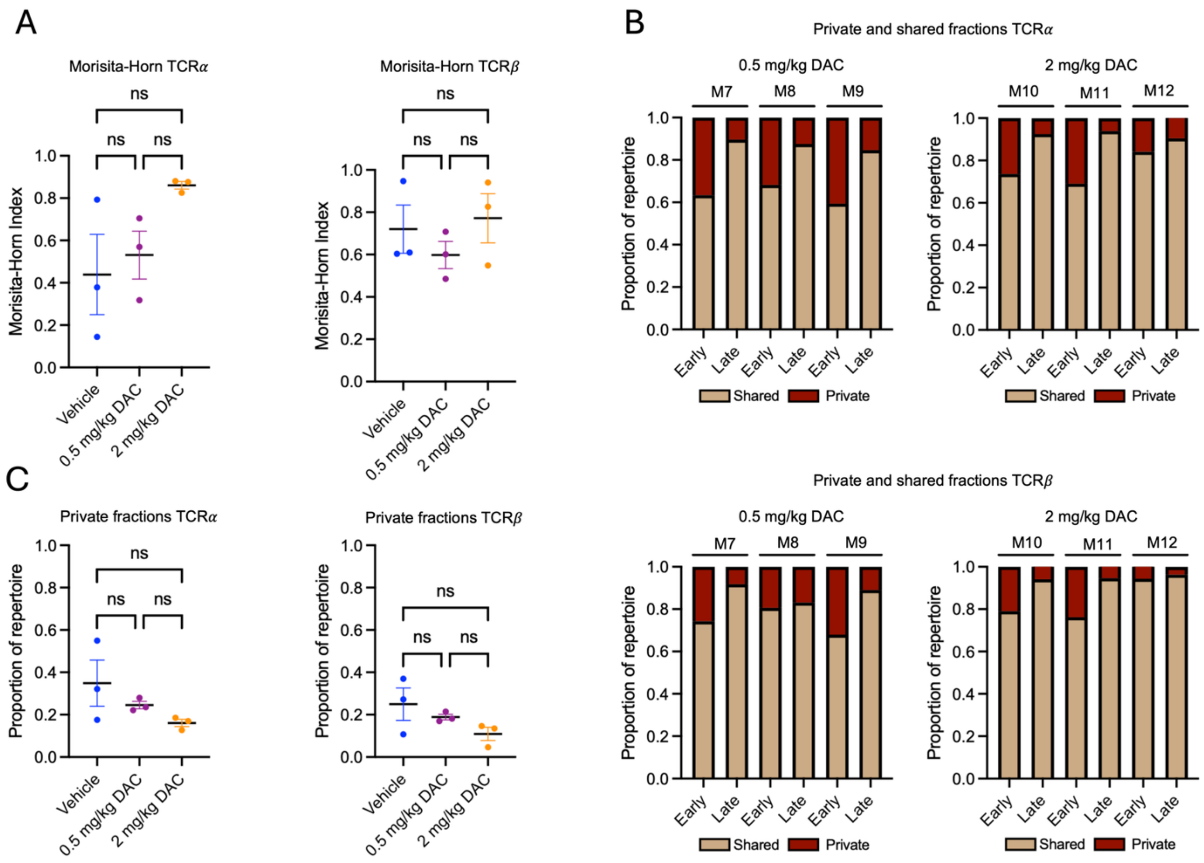

Supplementary Figure 4. Temporal repertoire remodeling remains consistent across DAC treatment conditions for both TCRα and TCRβ. A) Morisita–Horn similarity indices comparing paired early and late tumors within individual mice across treatment groups for TCRα (left) and TCRβ (right) (M7-9 & M10-12). **B)** Proportion of shared and private clonotypes in mice treated with 0.5 mg/kg DAC (left) and 2 mg/kg DAC (right) for TCRα (upper panels) and TCRβ (lower panels). **C)** Proportion of private clonotypes within individual mice across treatment groups for TCRα (left) and TCRβ (right). Data are presented as mean ± SEM. Statistical significance was assessed using one-way ANOVA with Tukey's multiple comparisons test. ns, $p > 0.05$. N = 3 mice per group.
